# An Experimental Study on the Influence of Different Cooling Methods on the Mechanical Properties of PVA Fiber-Reinforced High-Strength Concrete after High-Temperature Action

**DOI:** 10.3390/polym16142012

**Published:** 2024-07-14

**Authors:** Jian Wu, Chaoqun Hu, Yuxi Wang, Liangjie Hu, Lidan Zhang, Jianhui Wang, Weigao Ding

**Affiliations:** 1 Shaanxi Key Laboratory of Safety and Durability of Concrete Structures, Xijing University, Xi’an 710123, China; wujian2085@126.com (J.W.);; 2 School of Infrastructure Engineering, Dalian University of Technology, Dalian 116024, China

**Keywords:** PVA fiber, HSC, high temperature, cooling method, mechanical and physical properties

## Abstract

High-strength concrete (HSC) has a high compressive strength, high density, excellent durability, and seepage resistance, but its deformation ability is weak. Adding fibers can improve the physical and mechanical properties of HSC. Additionally, the HSC structure may face the threat of fire. In the process of fire extinguishing, the damage mechanism of high-temperature-resistant concrete is complicated due to the different contact conditions with water at different locations. Hence, it is essential to conduct pertinent research on the behavior of fiber-reinforced HSC with different cooling methods after high-temperature action. In this paper, polyvinyl alcohol fiber (PVA fiber) was selected to be added into the HSC to carry out high-temperature experimental research, so as to explore the apparent changes, failure pattern, and mass loss rate of the fiber-reinforced HSC using different cooling methods and analyze the influence of its residual compressive strength and flexural strength. The test results suggest that, with the increase in heating temperature, the color of the specimen’s surface transitions from dark blue-gray to white, and the quantity of surface cracks on the specimen gradually rises. The mechanical strength gradually decreases as the heating temperature increases. At a consistent heating temperature, the mechanical strength initially rises, and then falls with an increase in fiber content. The maximum compressive strength and flexural strength were achieved at PVA fiber contents of 0.2% and 0.3%, respectively. For different temperatures and fiber contents, the mechanical strength after natural cooling is generally higher than that after immersion cooling. In addition, X-ray polycrystalline diffractometry (XRD) and scanning electron microscopy (SEM) tests were conducted to analyze the compositional alterations and microstructure of the fiber-reinforced HSC following high-temperature exposure, accompanied by an explanation of the factors influencing the alterations in the physical and mechanical properties. Therefore, the findings of this study can serve as a valuable reference for the utilization of HSC in engineering structures and contribute to the advancement of HSC technology.

## 1. Introduction

High-strength concrete (HSC) is a type of concrete characterized by high strength, high compression resistance, and excellent durability. By adding a certain amount of a high-efficiency water-reducing agent or a certain amount of active mineral materials at the same time, it is made via an ordinary molding process and has high-strength properties [1,2,3,4]. Therefore, it has been extensively utilized in high-rise building structures and long-spanning bridge structures. However, HSC is prone to bursting at high temperatures, and the greater the concrete strength grade is, and the higher the temperature it is subjected to, the higher the probability of the burst phenomenon occurring is, and its damage degree will increase. Therefore, Klingsch [5] chose to add fiber into concrete to improve the high-temperature cracking phenomenon of HSC. The mechanical properties of fiber-reinforced HSC are affected not only by temperature, but also by the way of cooling after high-temperature action. After a building catches on fire, in the process of extinguishing the fire, because of the considerable height of a high-rise building structure, it is outside of the spray range, so the cooling method is similar to natural cooling. In a shorter, closed structure, only immersion cooling can be used to extinguish a fire. Therefore, it is important to study the properties of HSC exposed to high temperatures and subjected to various cooling effects.

The properties of HSC are strongly correlated with the water–binder ratio, the aggregate particle size, the admixtures, and the mineral admixtures. Mehta et al. [6] suggested that high-strength aggregate varieties with a high elastic modulus and low coefficient of thermal expansion should be used when preparing ultra-HSC. In the case of a given water–cement ratio, reducing the maximum grain diameter of the coarse aggregate could significantly enhance the strength of HSC. In accordance with a study by Aitcin et al. [7], the higher the target strength is, the smaller the maximum grain diameter of the coarse aggregate should be. To attain a compressive strength of 100 MPa, the concrete must employ a coarse aggregate with a maximum particle size falling within the range of 14 to 20 mm. For fine aggregates, it is recommended to use fine aggregates with a high fineness modulus (about 3.0). Because there are already a large number of fine particles of cement and volcanic ash in HSC, very fine particles in fine aggregates are not required to improve workability. At the same time, to decrease water usage in concrete mixing, improve its pore structure, and increase the compactness, a high-efficiency water-reducing agent could be added to HSC. A carboxylic acid admixture is a kind of concrete admixture with a high water reduction rate and minimal slump loss, which could greatly increase the rheological property of new mixed concrete under the condition of constant water consumption [8]. In terms of the mix ratio, the amount of cementing material in HSC is higher, generally greater than 400 kg/m^3^. Aldahdooh et al. [9] have studied cases where the amount of cementing material is 600 kg/m^3^ or even higher, but this practice is not advisable because of the high cost and excessive temperature shrinkage. At present, the mineral admixtures of concrete are mainly fly ash, granulated blast furnace slag, and silica fume. The main active component of fly ash and silica fume is SiO_2_, and Ca(OH)_2_ produced by the reaction will produce a hydrated calcium silicate gel after reacting with volcanic ash. This colloid is filled between the pores of the aggregate, resulting in a denser concrete, which could effectively improve its durability [10]. The above literature has basically determined the influence of parameters such as the aggregates, minerals, cement content, and water–cement ratio on the performance of high-strength concrete. However, ordinary mix proportions can easily lead to high costs. Therefore, this paper adds water-reducing agents in the manufacturing process of HSC.

With the increase in the strength of HSC, its brittleness is more obvious, its strain-softening property is poor under axial pressure, and it undergoes sudden burst failures. The addition of fibers is a successful approach to enhance the toughness and crack resistance of HSC. Klingsch [5] incorporated polypropylene fibers (PPFs) into HSC to control the volume changes in concrete during rapid temperature fluctuations and high-temperature conditions, thereby minimizing the formation and propagation of micro-defects in the concrete. Mugume et al. [11] found that the bursting degree of HSC specimens with a length of 12 mm of PVA fiber was much smaller than the HSC specimens with a length of 6 mm. Caetano et al. [12] found that PVA fiber (PVAF) had a binding effect on concrete and could inhibit the development of cracks. Xiao et al. [13] conducted a study and the analysis of the axial compression mechanical characteristics of steel–PVA mixed concrete short columns under high temperatures. The findings demonstrated that the incorporation of PVA fibers could successfully diminish the development and propagation of surface cracks in the columns, while also improving their ductility. Li et al. [14] found that the tensile strength of the PVA fiber-reinforced concrete (FRC) specimen mixed with a 2.0% volume rate of PVA fiber was 4.5 Mpa, and the ultimate tensile strain exceeded 4%. Moreover, the surface of the specimen showed a failure pattern of multiple cracks, showing a good toughness that the ordinary, plain HSC did not have. At present, there is more research on synthetic fiber-reinforced concrete, such as PPF and PVAF, while less research on PVA fiber-reinforced concrete has been conducted, especially PVA fiber-reinforced HSC. PVA fiber is a type of synthetic fiber known for its high strength and elastic modulus, as well as excellent hydrophilicity. It can absorb a small quantity of free water on its surface and exhibits strong bonding with the cement matrix [15]. Zhang et al. [16] investigated the influence of fiber content and temperature on the mechanical strength and microstructure of metakaolin-/fly ash-based geopolymer mortar. The results showed that the mechanical strength decreased as the temperature became too high, and more cracks and pores appeared. Mpalaskas et al. [17] used the ultrasonic method and the acoustic emission method to analyze the influence of temperature on the cracking phenomenon of materials. Voutetaki et al. [18] studied the application of structural health monitoring (SHM) on the non-destructive inspection of fiber-reinforced concrete. The results showed that the root-mean-squared deviation was reliable in measuring structural damage. Thus, PVA fiber can effectively inhibit early-stage plastic cracking in concrete and improve its toughness and impact resistance [19]. These research results demonstrate that the addition of fibers can limit the cracking of ordinary concrete and improve the mechanical properties and microstructure of concrete to a certain extent. However, the application of fibers in high-strength concrete is still relatively limited, so this paper chooses PBA fiber-reinforced high-strength concrete as the research object.

At the same time, the temperature of the concrete in different parts is different in the firefighting process for engineering structures, and the contact with water is also different, which leads to the complex damage mechanism of the concrete after the action of fire. Researchers have carried out relevant experimental research to investigate the impact of high temperature and the cooling method on the properties of HSC. Chan [20] and Morsy [21] found that high temperatures would cause the decomposition of carbonizable substances inside concrete, and the voids generated after the decomposition would reduce the strength of the concrete. Peng et al. [22] studied and analyzed the impact of different cooling methods on the residual mechanical properties of fiber-reinforced concrete treated at 200 °C~800 °C. The results indicated that, compared to natural cooling, concrete cooled using the water spraying method suffered more severe damage. Luo et al. [23] studied the residual compressive strength of high-performance concrete (HPC) using two cooling methods (natural cooling and spray cooling) at 800 °C and 1100 °C. The results showed that the residual compressive strength of HPC at high temperature decreases sharply compared with room temperature. Compared with natural cooling, spray cooling produced significant thermal shock, resulting in a more serious strength decline. An et al. [24] conducted dynamic compression tests on basalt fiber-reinforced concrete with different loading rates, heating temperatures, and cooling methods. The findings indicated that, as the temperature increased, the mass loss and wave velocity decreased, and the rate of decrease in the strength under natural cooling surpassed that under water cooling action. Existing research demonstrates that water cooling significantly impacts the behavior of concrete after high-temperature action. However, due to the uneven distribution and the complex change of the internal temperature field under high-temperature circumstances, and the difference of the mechanical properties of concrete under different high temperatures, the research results are even more different. Elsalam et al. [25] studied the properties of glass fiber-reinforced concrete (GRC) columns under the action of axial loads and high temperature. The results showed that the fire resistance of GRC columns was significantly affected by the axial loads. These articles have studied the performance changes and microstructure of ordinary concrete under different cooling systems after high-temperature action. However, high-strength concrete is denser and prone to cracking under high-temperature action. Therefore, it is necessary to consider the influence of temperature and cooling methods on the performance of high-strength concrete.

Therefore, the main research contents of this paper mainly include the appearance, mass loss rate, compressive strength, and flexural strength of specimens, and the calculation formulas of the strength and temperature using different cooling methods are proposed by regression analysis. Furthermore, the impact of heating temperatures on the composition of PVA fiber-reinforced HSC is analyzed by XRD and SEM tests, and the connection between the change of composition and macroscopic properties of PVA fiber-reinforced HSC is revealed. The research findings of this paper will help to explore the possible phenomenon of engineering structures using fiber-reinforced high-strength concrete under the high-temperature action and contribute to ensuring the safety and designing of structures.

## 2. Materials and Methods

### 2.1. Materials

In this paper, ordinary Portland cement with a strength grade of P·O 52.5 was chosen as the cementing material manufactured by Zhucheng Jiuqi Building Materials Co., Ltd. in Weifang, China. The particle size range of the coarse aggregate was 5~20 mm, and the ordinary river sand characterized by a fineness modulus of 2.63 and a packing density of 1480 kg/m^3^ was selected as the fine aggregate. In order to reduce the water–binder ratio, polycarboxylic acid superplasticizer with a water reduction rate of 30% produced by Shanxi Weike Building Materials Co., Ltd. in Changzhi, China, was added in the concrete configuration process. The mineral materials such as fly ash with an apparent density of 2600 kg/m^3^ and silica fume with a 7d activity index of 116% were also used to improve the performance of the HSC. The fiber used in this paper was produced by Shanghai Kaiyuan Chemical Technology Co., Ltd. in Shanghai, China, and the properties of the PVA fiber can be found in Table 1.

### 2.2. Specimen Design

#### 2.2.1. Mix Ratio

The strength grade of PVA fiber-reinforced HSC is C80; the water–binder ratio was established as 0.2 by the trial mixing method, and the mineral admixtures were incorporated to enhance the performance of the HSC [6]. The content of the PVA fiber was 0%, 0.1%, 0.2%, 0.3%, and 0.4%, respectively. The mix proportions of the HSC can be found in Table 2. In order to ensure the quality of the HSC, the concrete mixture was poured into the mold first, then the mold was put on the vibrating table for compaction, and finally, the surface of the concrete was smoothed. After removing the mold, the specimens were cured in the curing box with a curing temperature of 20 ± 1 °C and a humidity of 90% for 28 d.

#### 2.2.2. Dimension and the Number of the Specimens

In order to fully investigate the impact of PVA fiber content, heating temperature, and cooling method on the performance of HSC, this paper mainly uses cube and prismatic specimens to carry out the experimental research. According to the relevant test procedures and requirements in the Chinese standard CECS 13-2009 [26], the compressive strength tests used cubic specimens with dimension of 100 mm × 100 mm × 100 mm, while the flexural strength tests used prismatic specimens with dimension of 100 mm × 100 mm × 400 mm. The appearance, failure pattern, and mechanical properties of the specimens after high-temperature action were analyzed. Considering the variables (temperature, fiber content, cooling method, and test type) used in the experimental design of this paper, the number of specimens is given in Table 3. It should be noted that physical performance testing was conducted after the testing of the mechanical properties, so this would not increase the number of specimens.

### 2.3. Heating Equipment and High-Temperature Loading System

The high-temperature test was carried out with the LYL-16TL high-temperature trolley furnace produced by Luoyang Liyu Pit Furnace Co., Ltd. in Luoyang, China. The external size of the combustion furnace is 2100 mm × 1800 mm × 2000 mm; the maximum temperature is 1200 °C; the working voltage is 380 V; the temperature rise program can be set; the temperature in the furnace can be automatically controlled. When the predetermined heating temperature is reached, the system will automatically maintain a constant temperature, which is convenient for temperature controlling and recording. However, the development of fire in engineering structures is usually difficult to predict; thus, for the concrete in the structure, the actual fire temperature curves are diverse. In order to unify the evaluation criteria for easy comparison, many countries and organizations have developed standard fire temperature curves for the fire resistance test to assess the fire resistance of concrete. In China, the standard fire rising temperature curve for general industrial and civil buildings is consistent with the specifications of ISO 834-1:1999 [24,27], and the calculation Formula (1) of the curve is as follows:

(1) $$T_{g}-T_{g0}=345l_{g}(8t+1)$$

in which *t* represents the warming time (minute) and *T_g_* and *T_g_*_0_ are the average temperature of hot flue gas corresponding to time *t* and the temperature of the indoor environment before the fire broke out (°C), respectively.

**Figure 1:**
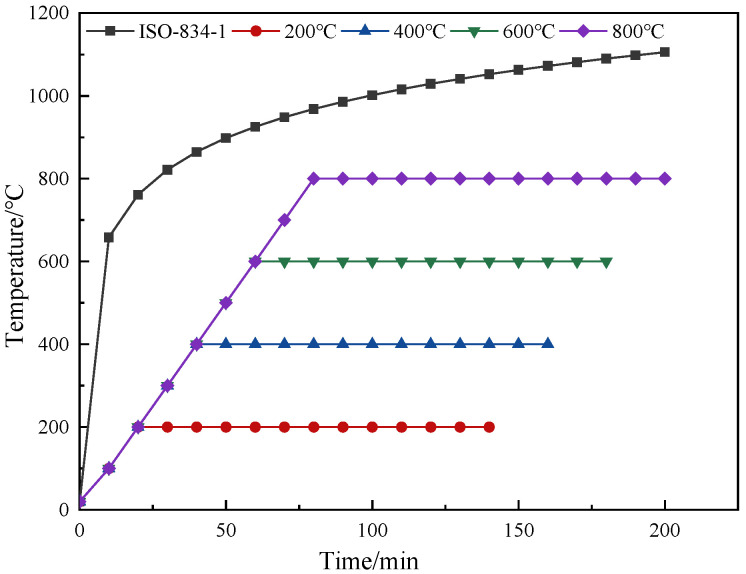
Standard temperature rising curve and actual temperature rising curve.

**Figure 2:**
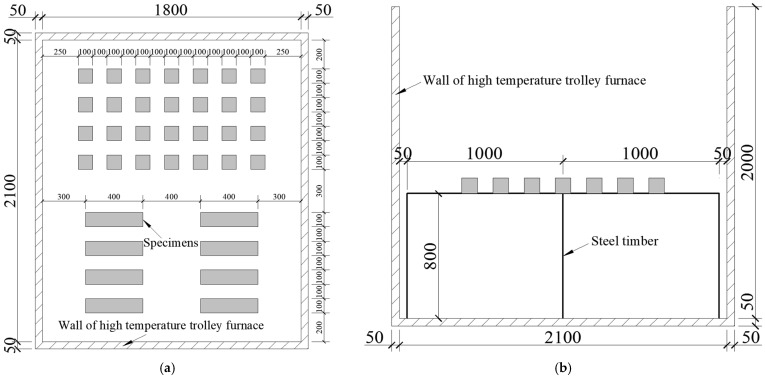
Layout diagram of the specimens: (**a**) vertical view; (**b**) side view.

Before the high-temperature test, all the samples were weighed and measured first, and then, the samples were heated in the high-temperature furnace; the heating speed was set to 10 °C/minute. When the specified heating temperature (200 °C, 400 °C, 600 °C, 800 °C) was reached, the temperature was maintained for 120 min to ensure that the temperature in the core area of the samples could also reach the preset temperature value. The standard and actual temperature rising curves are shown in Figure 1. In order to better ensure the uniformity of the heating of the specimens, only one layer of specimen was placed in the high-temperature furnace for each test, and the arrangement of the specimens is shown in Figure 2.

### 2.4. Cooling Methods

**Figure 3:**
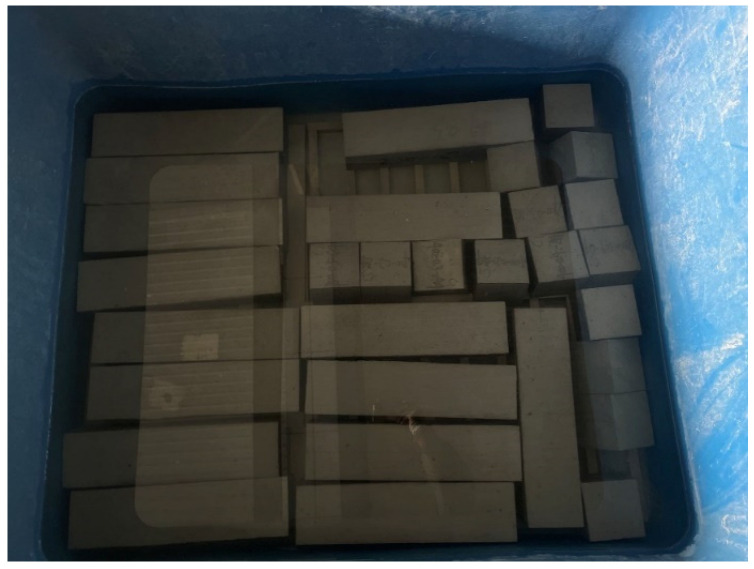
Immersion cooling diagram.

The fiber-reinforced HSC specimens after high-temperature treatment were separated into two groups. The initial group was placed in cold water for immersion cooling, that is the sample was immersed in a vessel filled with water, and the water level was maintained above the top surface of the specimens throughout the entire cooling process, as shown in Figure 3. After the specimens were cooled using different methods, the physical and mechanical performance tests were conducted after drying them in a natural environment until there was no significant change in quality.

## 3. Physical Properties of Specimens after High-Temperature Action

### 3.1. Apparent Characteristic Analysis

**Figure 4:**
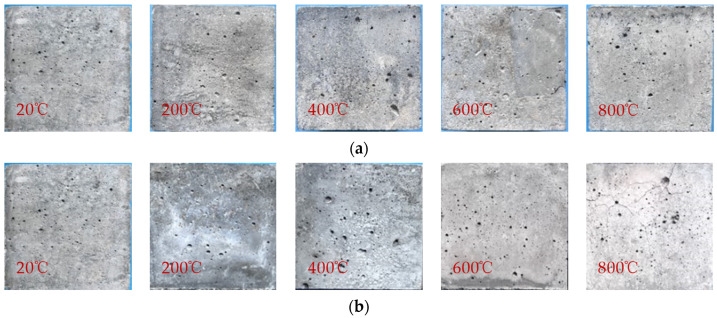
Apparent phenomenon of PVA fiber-reinforced concrete sample after high-temperature action: (**a**) natural cooling; (**b**) immersion cooling.

It could be observed that the fiber content had minimal impact on the color of the specimens. Therefore, this paper focuses on observing the specimens with fiber content of 0.2%, and the color changes of the specimens after the treatment of immersion cooling and naturally cooling were compared, as shown in Figure 4. At 20 °C and 200 °C, the color of the specimens under both cooling methods was bluish gray. When the temperature reached 400 °C, the surface color of the specimens cooled by natural cooling became slightly lighter, while for the specimens cooled by immersion cooling, the color became light yellow due to the reaction of internal impurities with water; moreover, fine cracks could be observed on the surface of the specimen. At 600 °C, Ca(OH)_2_ in the HSC would be dehydrated and decomposed, and the main components after natural cooling were CaO, SiO_2_, etc.; thus, the colors were gray and white. However, the surface color of the specimens cooled by immersion cooling turned to a dark brown-yellow color due to the rust reaction of the composition in the HSC. At 800 °C, the gel inside the concrete underwent nearly complete transformation, and the surface of the specimens appeared white under the two cooling methods. At the same time, the cracks gradually developed and the width of the cracks increased.

### 3.2. Mass Loss Rate

When the specimens are subjected to high temperatures, the internal moisture and volatile substances will evaporate or decompose, leading to the reduction of specimen mass. The ratio of reduced mass to the original mass is referred to as the mass loss rate. The composition, structure, and calcination temperature of concrete are the factors that influence the mass loss rate. To explore the quality changes of PVA fiber-reinforced HSC, the mass of the samples before and after the high-temperature tests was measured by an electronic balance, and the mass loss rate of the samples in each category can be computed following Equation (2).

(2) $$M_{r}=\frac{P_{0}-P_{t}}{P_{0}}\times 100\%$$

in which *M_r_* represents the mass loss rate (%) and *P*_0_ and *P_t_* are the mass of the specimen before and after high-temperature action (kg), respectively.

#### 3.2.1. Mass Loss Rate of Specimens Using Natural Cooling after High-Temperature Action

According to the quality change of specimens before and after the high-temperature tests, the mass loss rate of the specimens can be seen in Table 4. Also, the standard deviation (SD) and coefficient of variation (CV) are given in the table. It should be pointed out that the specimens were made in winter and are greatly affected by a low temperature. Meanwhile, the high-temperature and cooling process have a significant impact on the performance of the HSC, resulting in a relatively large value of the CV. Although it is generally believed that the limit value of the CV is 15%, considering the complexity of the specimen production and testing process, the results are still basically reliable.

**Figure 5:**
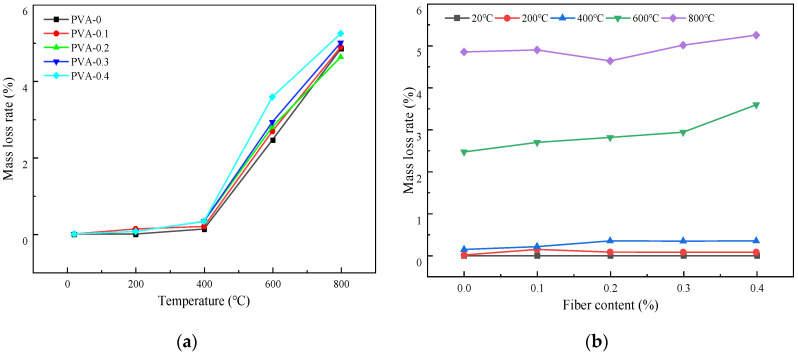
Variation of mass loss rate of samples using natural cooling method: (**a**) the change of mass loss rate with different temperatures; (**b**) the change of mass loss rate with different fiber contents.

Figure 5 shows the relationship curve of the mass loss rate with different heating temperatures and fiber contents under the natural cooling treatment.

Figure 5a gives the change of the mass loss rate of the samples with different temperatures under the natural cooling condition. It is evident from the figure that the mass loss rate keeps increasing as the temperature increases. At 400 °C, the mass loss rates of specimens with a fiber content of 0% to 4% were 0.132%, 0.200%, 0.335%, 0.332%, and 0.336%, respectively. When the temperature increased to 600 °C, the mass loss rates were 2.463%, 2.692%, 2.808%, 2.935%, and 3.589%, respectively. In the temperature range of 400 °C~600 °C, the mass loss rate for each fiber content increased by 17.6 times, 12.46 times, 7.38 times, 7.84 times, and 9.68 times, respectively. This phenomenon may be due to the fact that the temperature of 400 °C has exceeded the thermal decomposition temperature of the PVA fiber; thus, the rapid decomposition of the fiber and the release of volatile gas will occur [28]. Simultaneously, the evaporation rate of free water and bound water in concrete will be faster, and the hydration products of cement will be dehydrated and decomposed. Due to the release of water and other volatile substances, the mass of the concrete specimens decreased [29].

Figure 5b illustrates the variation in the mass loss rate of the samples with different fiber contents under the natural cooling condition after the high-temperature action. The figure demonstrates that, when the range of temperature varied between 20 °C and 400 °C, the mass loss rate of the specimens with different fiber contents remained below 0.4%. It can be concluded that the content of PVA fiber at a low temperature has little impact on the mass loss rate. When the temperatures ranged from 400 °C to 800 °C, the addition of fiber could improve the mass loss rate of the HSC. At room temperature, the increase in fiber content had little effect on the mass loss rate of the HSC. However, when the temperature was higher than 400 °C, the mass loss rate showed a trend of initially decreasing and then increasing. For all the specimens under the action of different temperatures, the smallest mass loss rate value would be obtained when the fiber content is 0.2%. It should be noted that, when the temperature was 800 °C, the mass loss rate decreased for the specimens with a fiber content of 0.2%. This phenomenon cannot be explained by the research in this paper at present and requires further in-depth analysis in the future.

#### 3.2.2. Mass Loss Rate of Specimens Using Immersion Cooling after High-Temperature Action

The results of the mass loss rate of the specimens using the immersion cooling method are given in Table 5. It is important to mention that the specimens without fibers were impaired at temperatures of 600 °C and 800 °C; thus, there are no experimental data at the corresponding position in the table.

**Figure 6:**
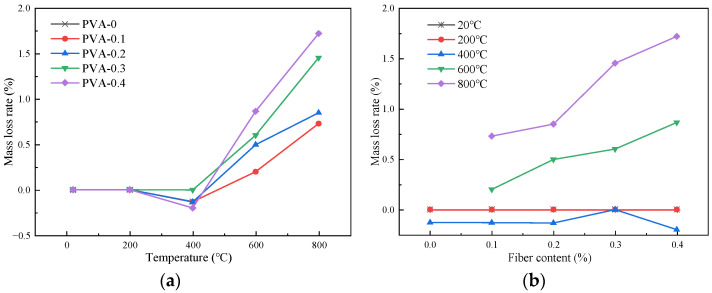
Variation of mass loss rate of samples using immersion cooling method: (**a**) the change of mass loss rate with different temperatures; (**b**) the change of mass loss rate with different fiber contents.

Figure 6 gives the relationship curve of the mass loss rate with different heating temperatures and fiber contents under the action of immersion cooling. As mentioned before, when heated to about 600 °C, the plain HSC will crack, and its physical and mechanical properties cannot be tested.

Figure 5a illustrates the change of the mass loss rate of the samples with different temperatures under the immersion cooling condition. At 200 °C, the mass loss rates of the specimens with different fiber contents were 0%. At 400 °C, the values for the specimens with fiber contents of 0, 0.1%, 0.2%, 0.3%, and 0.4% were −0.131%, −0.132%, −0.135%, −0.002%, and −0.201%, respectively. It can be concluded from the data that there were different degrees of negative growth. At 600 °C, the mass loss rate value increased rapidly. In short, the mass loss rate of the specimen using the immersion cooling method decreased first and then increased as the temperature increased. This phenomenon occurred because the HSC was cooled by immersion after the high-temperature action, and the absorbed mass exceeded the mass loss at high temperatures, leading to a decrease in the mass loss rate [28]. When the heating temperature was higher than 400 °C, the mass loss rate steadily increased with the increase in the temperature. This may be because the water absorption capacity of the HSC after the high-temperatures action was lower than the mass loss of the specimens.

Figure 5b presents the change of the mass loss rates of the samples with different fiber contents under the immersion cooling condition after the high-temperature action. It is evident from the figure that, at the temperatures of 20 °C and 200 °C, the mass loss rates of all the specimens were 0%. When the temperature was between 200 °C and 400 °C, the mass loss rate values decreased, but the change was not obvious. When the temperature was 600 °C and 800 °C, the mass loss rate values enhanced with the increase in the fiber content. This is because the PVA fiber will undergo a thermal decomposition reaction at high temperature. The greater the temperature is, the swifter the rate of thermal decomposition reaction is; thus, the mass loss rate will increase accordingly [30].

In summary, with the increase in temperature, the mass loss rate of the HSC showed a slow rising trend when the temperature was 200 °C~400 °C. The growth rate accelerated significantly at 400 °C and 600 °C, and the values under the natural cooling treatment were greater than that of immersion cooling. At 800 °C, the value of naturally cooled concrete was 4.9%, while that of the immersed cooling specimen was 1.2%. Under different cooling treatments, the mass loss rate of the specimen increased with the change of fiber content. At 400 °C~800 °C, the mass loss rate of the fiber-reinforced concrete under natural cooling was much larger than that under the action of immersion cooling [22].

## 4. Mechanical Properties of Specimens after High-Temperature Action

### 4.1. Compressive Strength after High-Temperature Action

The compressive strength of the samples after the high-temperature action is collectively referred to as residual strength *f_c_*. The loading tests were conducted using an electro-hydraulic servo universal testing machine. The testing process involved consistent and uninterrupted loading with a loading rate of 0.8 MPa/s. The experiment was conducted according to the relevant provisions of the Chinese standard GB/T 50081-2019 [31]. The compressive strength should be determined using Equation (3).

(3) $$f_{c}=\frac{F}{A}$$

in which *f_c_* represents the compressive strength of the specimen (MPa), *F* is the ultimate load of the specimen (N), and *A* is the bearing area of the specimen (mm^2^).

The compressive strength test results of the specimens with different PVA contents after different high-temperature actions and cooling treatments are given in Table 6.

#### 4.1.1. Influence of Fiber Content on the Compressive Strength

**Figure 7:**
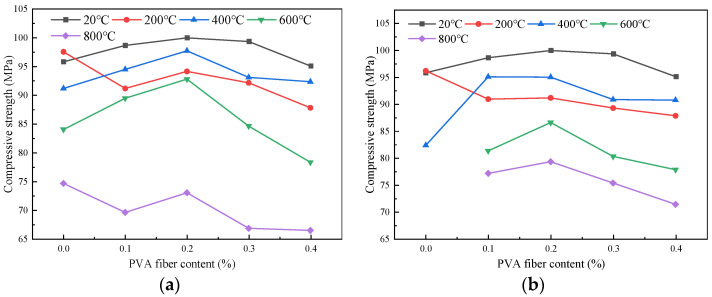
Influence curve of PVA content on compressive strength of specimens: (**a**) natural cooling; (**b**) immersion cooling.

The impact of fiber content on the compressive strength of the specimens after high-temperature action is given in Figure 7. It could be observed that, under natural cooling conditions, the compressive strengths of the specimens with 0.1% fiber content at different temperatures (20 °C, 200 °C, 400 °C, 600 °C, and 800 °C) were 98.64 MPa, 91.17 MPa, 94.5 MPa, 89.46 MPa, and 69.57 MPa, respectively. When the fiber content was 0.2%, the compressive strength increased by 1.35 MPa, 2.97 MPa, 3.24 MPa, 3.33 MPa, and 3.42 MPa, respectively. When the fiber content was 0.3%, the compressive strength of the samples decreased by 0.63 MPa, 1.98 MPa, 4.63 MPa, 8.19 MPa, and 6.21 MPa, respectively. Under the condition of the same temperature, the compressive strength of the concrete initially increased and then decreased. The maximum strength value of specimens at different temperatures under different cooling methods would be obtained when the fiber content is 0.2%. This is because an appropriate amount of fiber can decrease the expansion coefficient of the concrete. The visible indication is that, when the fiber content is not higher than 0.2%, the surface of the specimens develop extensive cracks after exposure to high temperatures; conversely, for the specimens with a fiber content exceeding 0.2%, the fluidity of the concrete decreased with the increase in the fiber content. At the same time, more pores could be observed on the surface of the specimens, which affected the strength of the concrete [14]. The relationship between the mechanical strength and microstructure will be discussed in Section 5.

#### 4.1.2. Influence of Heating Temperature on the Compressive Strength

**Figure 8:**
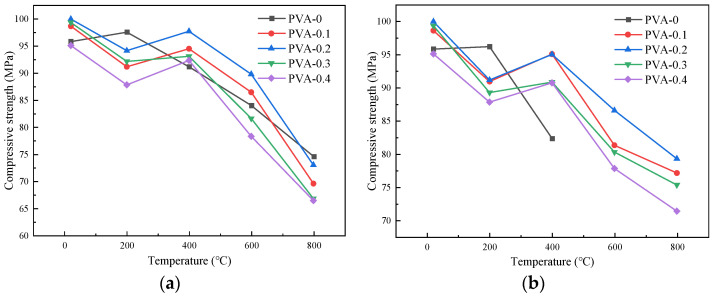
Influence curve of heating temperature on compressive strength of specimen: (**a**) natural cooling; (**b**) immersion cooling.

Figure 8 shows the influence of the heating temperature on the compressive strength of the specimens. When the temperature was 20 °C, the compressive strength of the plain HSC using the two cooling methods was 98.85 MPa. At a temperature of 200 °C, the compressive strength of the plain HSC under the two cooling conditions increased by 1.71 MPa and 0.36 MPa, respectively. This was due to the dense internal structure of the HSC promoting the hydration of cement particles at a low temperature, while the increase in hydration products led to the increase in strength.

For the samples mixed with PVA fiber, the change of compressive strength exhibited a trend of initially decreasing, then increasing, and finally, decreasing with the increasing temperature under different cooling treatments. Taking natural cooling as an example, at 200 °C, the compressive strengths of the specimens with fiber contents of 0~0.4% were 91.17 MPa, 94.14 MPa, 92.16 MPa, and 87.80 MPa, respectively. At 400 °C, the compressive strengths were 94.5 MPa, 97.74 MPa, 93.11 MPa, and 92.34 MPa, respectively. This is due to the PVA fiber melting as the temperature increased, causing the temperature of the outer surface to be higher than the interior, and the high temperature steam curing state was formed inside, which made the hydration reaction form cement stone and led to the increase in strength. In addition, silica fume and slag can fully fill the pores of the cement stone to make the concrete structure denser [32], offsetting the strength loss caused by external structural changes, thus generally improving the strength. When the temperature was 600 °C, Ca(OH)_2_ generated by cement hydration was decomposed, and the compressive strength decreased significantly. At 800 °C, the compressive strength remained about 70% of the original value. The change of the compressive strength after immersion cooling treatment was similar to that of natural cooling.

#### 4.1.3. Influence of Cooling Method on the Compressive Strength

**Figure 9:**
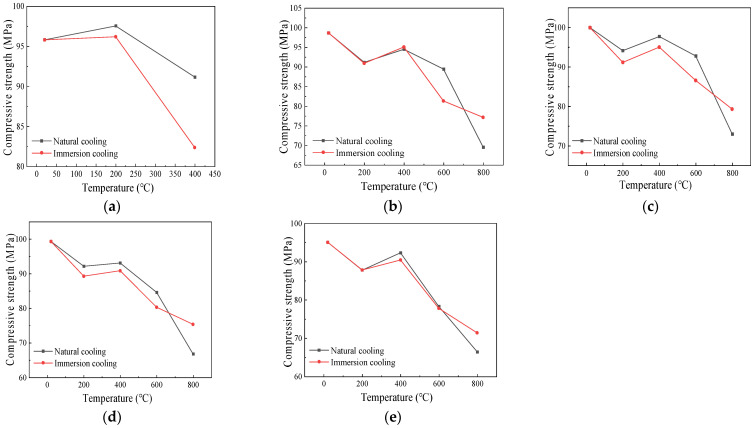
Influence curve of cooling method on compressive strength of specimens with different fiber contents: (**a**) 0%; (**b**) 0.1%; (**c**) 0.2%; (**d**) 0.3%; (**e**) 0.4%.

Figure 9 depicts the variation in the compressive strength of the specimens at different temperatures under the action of the two cooling methods. The compressive strength of the specimens without fibers initially increased and subsequently decreased as the temperature increased. However, the compressive strength of the specimens with the addition of fibers decreased when the temperature increased to 200 °C. This occurred because the fibers gradually melted as the temperature increased, leading to an increase in the internal pores of the specimens and a decrease in the compactness, consequently reducing the compressive strength [8].

Specifically, when the heating temperatures were 20 °C~600 °C, taking specimens with a fiber content of 0.2% as an example, the compressive strengths under the action of natural cooling were 99.99 MPa, 94.14 MPa, 97.74 MPa, and 92.79 MPa, respectively. The compressive strengths using the immersion cooling method were 99.99 MPa, 91.17 MPa, 95.04 MPa, and 86.58 MPa, respectively. The compressive strength under the natural cooling condition exceeded that using the immersion cooling method. This is because immersion cooling would rapidly reduce the temperature of the exterior of the specimens, while the internal temperature would still be higher, resulting in the temperature stress inside the specimen, which progressively increased as the temperature increased. Cracks appeared inside the concrete and gradually developed outward. Therefore, the decrease in compressive strength with the immersion cooling treatment was greater than that of the concrete using the natural cooling method [33]. From 600 °C to 800 °C, the compressive strength cooled by immersion in water decreased slowly, because the main components after the decomposition of the specimens were CaO and SiO_2_, which could form hydrated crystals such as Ca(OH)_2_ in water, thus enhancing the compressive strength. For the specimens with the natural cooling treatment, the compressive strength decreased rapidly due to the thermal breakdown of Ca(OH)_2_ resulting from cement hydration.

#### 4.1.4. The Correlation between Compressive Strength and Heating Temperature

The relationship between the compressive strength and the heating temperature after the high-temperature action was established under the treatment of the two different cooling methods. This was performed to simplify the implementation of the governing of the variation in the compressive strength with the temperature.

**Figure 10:**
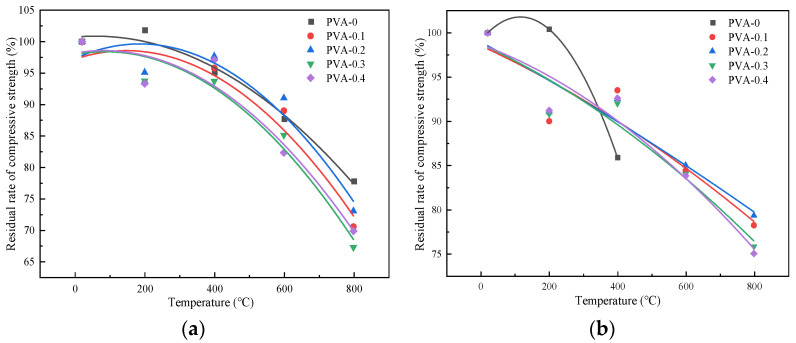
Fitting curve graph of compressive strength: (**a**) natural cooling method; (**b**) immersion cooling method.

The fitting curves of the compressive strength and heating temperature of the specimens after the high-temperature action are shown in Figure 10.

Under the action of the two cooling methods, the variation in the compressive strength of the specimens with different fiber contents and heating temperatures can be approximated by Equation (4):

(4) $$\frac{f_{cu}^{T}}{f_{cu}}=A+BT+CT^{2}$$

where *T* is the temperature (20 °C ≤ *T* ≤ 800 °C), *A*, *B*, and *C* are unknown parameters in the formula, and $f_{cu}^{T}$ and $f_{cu}$ are the compressive strengths of the specimen after the high-temperature action (MPa), respectively.

Specific values are given in Table 7.

### 4.2. Flexural Strength after High-Temperature Action

The flexural strength of concrete can not only reflect the bending performance of the HSC, but can also reflect the toughening effect of the fiber. The experiments were conducted according to the specifications of the Chinese standard GB/T 50081-2019 [26]. Prismatic specimens with dimensions of 100 mm × 100 mm × 400 mm were used to obtain the flexural strength. The testing procedure adopted uniform and continuous loadings, and the loading speed was 0.8 MPa/s. The flexural strength of the specimens can be calculated by Equation (5):

(5) $$f_{t}=\frac{Fl}{bh^{2}}$$

in which *f_t_* is the flexural strength of the specimen (MPa), *F* is the ultimate load in the testing process (N), *l* is the span length between the supports (mm), and *b* and *h* are the section width and height of the specimen (mm), respectively.

The results of the flexural strength tests of the specimens with different fiber contents after the action of the different temperatures and cooling treatments are given in Table 8.

#### 4.2.1. Influence of Fiber Content on Flexural Strength

**Figure 11:**
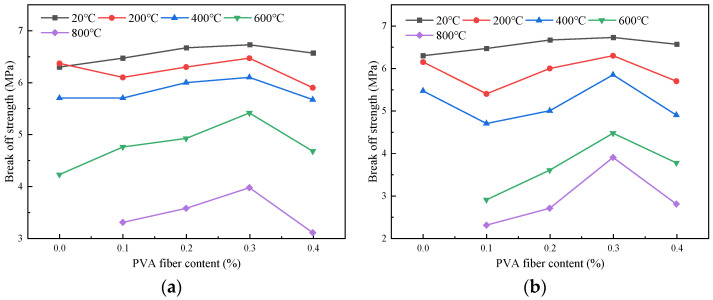
Influence curve of PVA content on flexural strength of specimen: (**a**) natural cooling; (**b**) immersion cooling.

The impact of the fiber content on the flexural strength of the specimens after the high-temperature action is shown in Figure 11. The flexural strength of the specimens with a fiber content of 0.1%, 0.2%, 0.3%, and 0.4% increased by 2.7%, 5.9%, 6.8%, and 4.3% compared with that of the plain HSC at room temperature. At a temperature of 200 °C, the flexural strength under the natural cooling treatment decreased by 4.2%, 1.1%, and 7.4%, respectively. When the fiber content was 0.3%, the value increased by 1.6% compared with the plain HSC. The highest flexural strength would be obtained when the fiber content is 0.3%. This may be due to the fact that, when the fiber content is small, the fibers cannot fully play their role, the dispersion density of fiber in the concrete is small, and the energy consumed by the pulling out of the fiber when the samples are stressed is less. Therefore, the flexural strength is relatively low from a macro perspective. However, when the fiber content increased, the dispersion density of the fibers increased; thus, the fiber was randomly distributed in the matrix and closely bonded to the matrix, and the fibers could better play their bridging role. Therefore, the flexural strength increased as the fiber content increased [34].

#### 4.2.2. Influence of Heating Temperature on Flexural Strength

**Figure 12:**
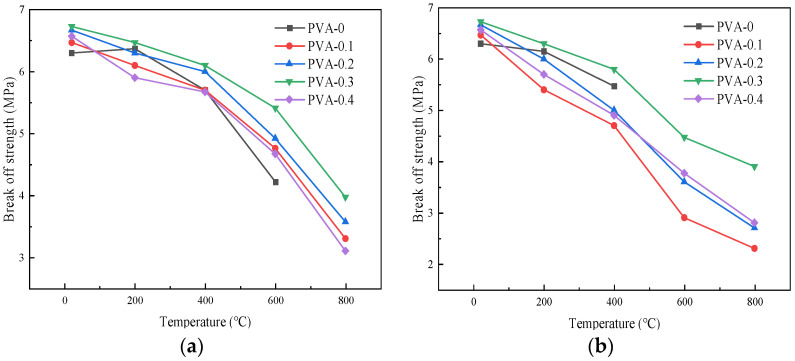
Influence curve of heating temperature on flexural strength of specimen: (**a**) natural cooling; (**b**) immersion cooling.

Figure 12 shows the influence of the heating temperature on the flexural strength of the specimens. Under the natural cooling conditions, at temperatures of 200 °C, 400 °C, 600 °C, and 800 °C, the flexural strength of the specimens with different fiber contents were 6.19 MPa, 5.87 MPa, 4.94 MPa, and 3.49 MPa, respectively. Compared to the specimens at room temperature, the flexural strength decreased by 6.4%, 11.2%, 25.3%, and 47.3%, respectively. It could also be observed that the flexural strength decreased by 6.4%, 4.8%, 14%, and 22%, respectively. Similarly, compared to the flexural strength of the specimens at room temperature, the flexural strength under the immersion cooling conditions decreased by 0%, 11.5%, 22.6%, 40.5%, and 55.8%, respectively. The flexural strength decreased as the heating temperature increased. This may be due to the reason that the high temperature caused the water in the HSC to evaporate and the structure inside the concrete to change, thereby reducing its flexural strength [35].

#### 4.2.3. Influence of Cooling Method on Flexural Strength

**Figure 13:**
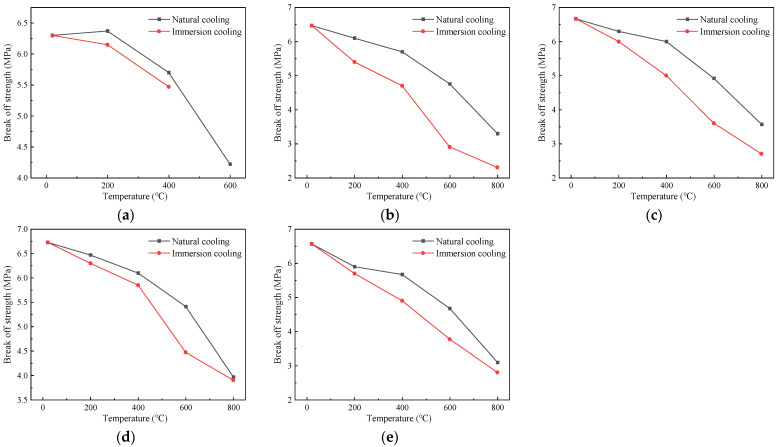
Influence curve of cooling method on flexural strength of specimens: (**a**) 0%; (**b**) 0.1%; (**c**) 0.2%; (**d**) 0.3%; (**e**) 0.4%.

Figure 13 illustrates how the flexural strength changes with different temperatures under the action of the two cooling methods. When the fiber content was 0.1%, the flexural strengths for each temperature under the natural cooling treatment were 6.47 MPa, 6.10 MPa, 5.70 MPa, 4.76 MPa, and 3.30 MPa, respectively. Under the action of immersion cooling, the flexural strengths for each temperature were 6.47 MPa, 5.40 MPa, 4.70 MPa, 2.90 MPa, and 2.30 MPa, respectively. For the specimens with a fiber content of 0.2%, the flexural strengths for each temperature under the action of natural cooling were 6.67 MPa, 6.30 MPa, 6.00 MPa, 4.92 MPa, and 3.57 MPa, respectively. Under the treatment of immersion cooling, the flexural strengths for each temperature were 6.67 MPa, 6.00 MPa, 5.00 MPa, 3.60 MPa, and 2.70 MPa, respectively. The flexural strength of the specimens after the action of different cooling methods gradually decreased in proportion to the increase in the temperature, and the strength values using the natural cooling method were greater than those using immersion cooling [23]. The reason for this phenomenon is that the cooling rate of the specimens was slow under the natural cooling action, which can better control the temperature distribution and stress distribution during the cooling process, thus reducing the internal stress and microcracks of the specimens [36].

#### 4.2.4. The Relationship between Flexural Strength and Heating Temperature

Under the action of the two cooling methods, the variation in the flexural strength of the specimens with different fiber contents and temperatures can be approximated by Equation (6):

(6) $$\frac{f_{f}^{T}}{f_{f}}=A+BT+CT^{2}$$

where *T* is the temperature (20 ℃≤T≤800 ℃), *A*, *B*, and *C* are unknown parameters in the formula, and $f_{f}^{T}$ and $f_{f}$ are the flexural strengths of the specimens after the high-temperature action (MPa), respectively.

**Figure 14:**
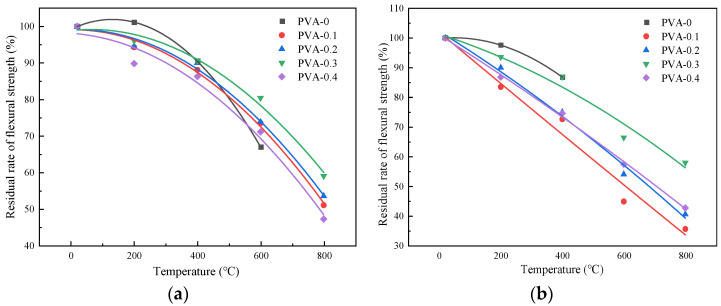
Fitting curve graph of flexural strength: (**a**) natural cooling method; (**b**) immersion cooling method.

The fitting curves of the flexural strength and heating temperature of the specimens after the high-temperature action are shown in Figure 14.

Specific values are given in Table 9.

## 5. Composition and Microstructure Analysis of Specimen after High-Temperature Action

### 5.1. Composition Analysis

#### 5.1.1. Principles of X-ray Diffraction Testing

X-ray energy spectrometry can analyze the elements in the scanning electron microscope qualitatively. The basic principle is to use an electron gun to emit high-energy electron beams to scan the surface of the samples, which makes the electron orbit within the atom transition and releases X-rays of certain energy. By detecting the energy of different photons, the elements in the samples can be identified [37].

#### 5.1.2. Test Method

When the specimens were damaged after mechanical testing, the core fragments were taken and repeatedly ground in a mortar. During the grinding process, it was necessary to ensure that the grinding force was consistent until there were no visible particles in the ground powder. After that, the powder should be classified and placed in a transparent sealed bag and marked properly.

#### 5.1.3. Analysis of Test Images and Results

**Figure 15:**
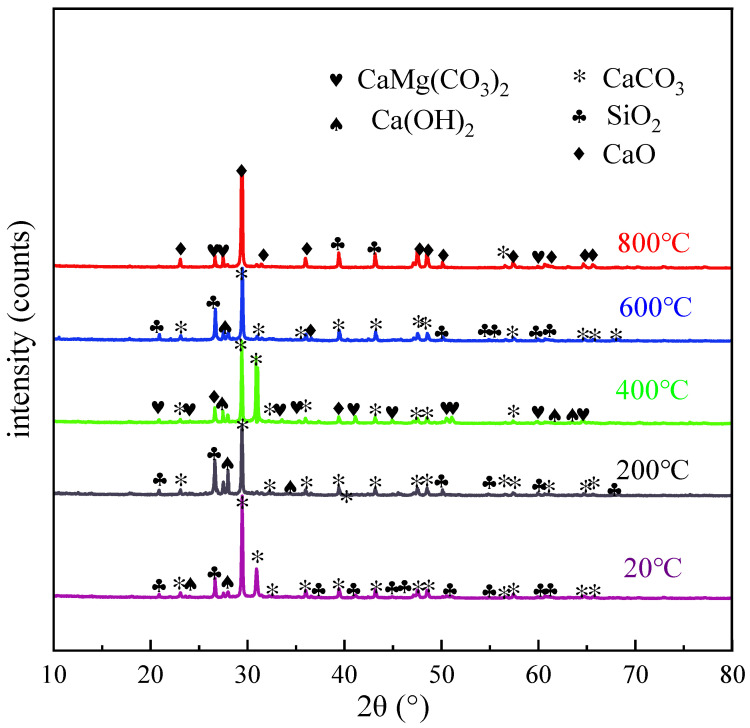
XRD patterns of HSC at different temperatures.

Figure 15 shows the XRD diffraction patterns of the specimens at different temperatures. As shown in the figure, the main material components of the concrete at a normal temperature were CaCO_3_, SiO_2_, and Ca(OH)_2_. In the figure, the content of CaCO_3_ and SiO_2_ is relatively high, while the content of Ca(OH)_2_ is relatively low. The change of the concrete phase composition after the high-temperature action can be divided into three stages: 20 °C~400 °C, 400 °C~600 °C, and 600 °C~800 °C. At temperatures of 20 °C~400 °C, the concrete mainly hydrated calcium silicate (C_3_S_2_H_4_, i.e., C-S-H), and calcium hydroxide (CH) began to decompose by dehydration. The dehydration process presented by Guirado et al. [38] is as follows:

(7) $$C_{1.62}SH_{1.5}\to \left[(1-\xi_{CSH})C_{1.62}SH_{1.5}+0.62\xi_{CSH}C_{2}S+0.38\xi_{CSH}CS\right]+1.5\xi_{CSH}H↑$$

(8) $$CH\to \left[\left(1-\xi_{CH}\right)CH+\xi_{CH}C\right]+\xi_{CH}H↑$$

in which $C_{1.62}SH_{1.5}$ represents the average composition of hydrated calcium silicate and $\xi_{CSH}$ and $\xi_{CH}$ are single-valued functions of the heating temperature.

When the temperature was between 400 °C and 600 °C, the height of Ca(OH)_2_ diffraction decreased. This is because the calcium hydroxide began to decompose when the temperature was higher than 400 °C, and calcium silicate hydrate also decomposed when the temperature reached 550 °C [39]. The specific processes are as follows:

(9) $$Ca{\left(OH\right)}_{2}\to CaO+H_{2}O$$

(10) $$CaMg{\left(CO_{3}\right)}_{2}\to CaO+MgO+2CO_{2}↑$$

At temperatures of 600 °C~800 °C, the diffraction strength of CaCO_3_ decreased continuously, indicating that its content gradually decreased after the high-temperature action, and the macroscopic observation indicated a decrease in compressive strength as the temperature increased. As the hydrated calcium silicate gel continued to dehydrate, Ca(OH)_2_ was decomposed into CaO, and CaCO_3_ and dolomite (CaMg(CO_3_)_2_) were decomposed. The equations are as follows:

(11) $$CaCO_{3}\to CaO+CO_{2}↑$$

The production of CO_2_ gas causes the texture of the concrete to become loose and the compactness to decrease, which could be verified by the fact that the compressive capacity of the concrete dropped sharply on the macro level. This experimental phenomenon is consistent with the results of other researchers [20,21].

### 5.2. Microstructure Analysis

#### 5.2.1. Composition Change of Concrete after High-Temperature Action

**Figure 16:**
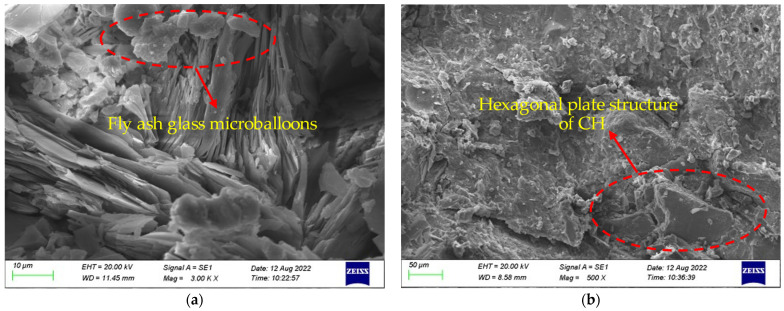

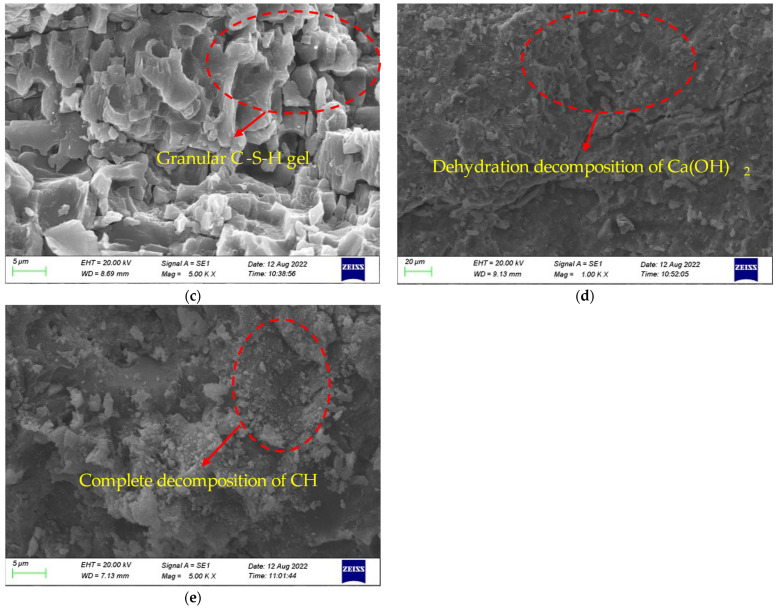
Composition change of high-strength concrete: (**a**) 20 °C, (**b**) 200 °C, (**c**) 400 °C, (**d**) 600 °C, and (**e**) 20 °C.

The change of composition under the action of different temperatures is given in Figure 16. At room temperature, the microstructure of the cement paste was complete, and unhydrated fly ash glass beads could be seen, as shown in Figure 16a. In Figure 16b, the C-S-H gel is decomposed, and the bound water and crystal water in the cement slurry were also decomposed by heat, while the microstructure began to show a loose state. When the temperature continued to increase, the cement paste showed obvious porosity and the number of microcracks increased significantly. However, at this time, all the cement was hydrated, and the newly generated hydration products improved the compressive strength of the concrete to a certain extent, which can be seen in Figure 16c. When the temperature was 600 °C, a large amount of Ca(OH)_2_ was dehydrated and decomposed, and the network structure of the gel was destroyed, as shown in Figure 16d. At this time, there were more cracks appearing, and the compressive strength decreased greatly. In Figure 16e, it can be seen that the cement paste was completely decomposed, and a large number of small cavities were generated inside the cement slurry. At this moment, the compressive strength decreased more obviously.

#### 5.2.2. Change of Appearance of Fiber-Reinforced HSC

**Figure 17:**
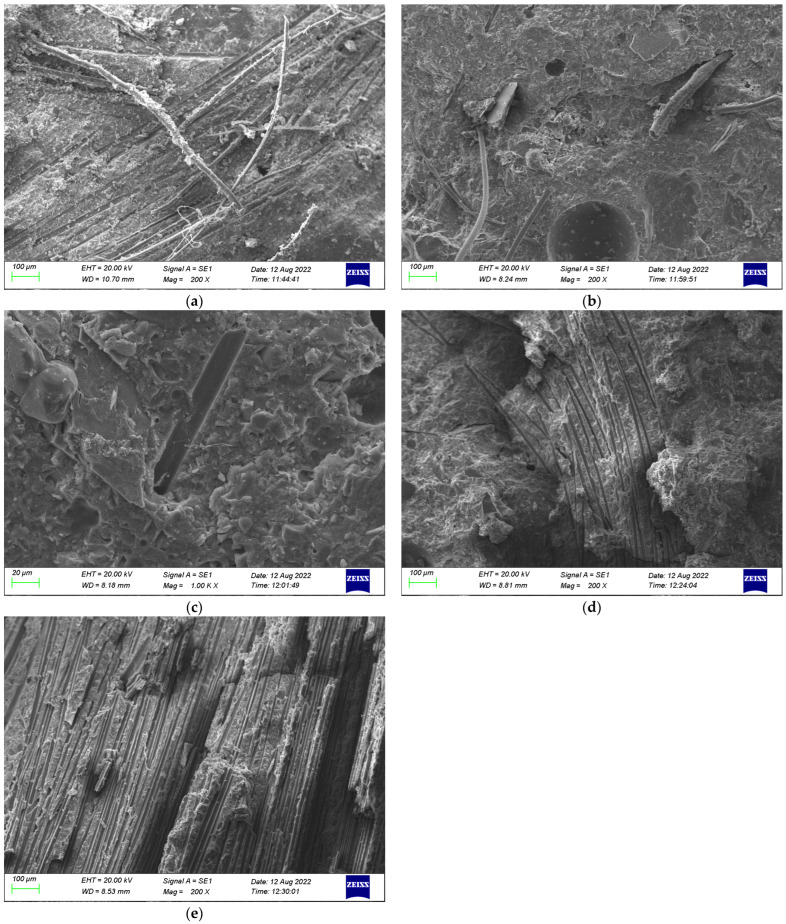
Microstructure change of HSC with fiber content of 0.2% under temperatures of (**a**) 20 °C, (**b**) 200 °C, (**c**) 400 °C, (**d**) 600 °C, and (**e**) 800 °C.

In this paper, the microstructural changes of the concrete under different temperature conditions were analyzed using a specimen with a fiber content of 0.2%, as given in Figure 17. At room temperature, the bonding performance between the fibers and concrete was relatively good (Figure 17a). At a temperature of 200 °C, although the fibers did not melt, gaps began to appear between the fiber and cement paste, as shown in Figure 17b. As the temperature continued to increase, the pores left by the melting of fibers could be seen in the SEM images, and cracks gradually began to appear in the cement paste (Figure 17c,d). When the temperature reached 800 °C, a large number of cracks began to appear in the cement paste (Figure 17e), and the macroscopic performance of the HSC showed a trend of a rapid decline in the mechanical properties.

## 6. Conclusions

In this paper, the specimens with different PVA fiber content were tested after high-temperature action to explore the physical and mechanical properties of the specimens under different cooling conditions. The effects of the temperature, PVA fiber content, and cooling method on the appearance, mass loss rate, compressive strength, and flexural strength of the specimens were analyzed. At the same time, the composition changes of the samples after high-temperature action were studied by XRD and SEM. The primary conclusions of this paper are outlined as follows:

(1) When the specimens were cooled in the natural environment, the color of the specimens was successively cyan-gray, light cyan-gray, gray-white, and white, while under the action of immersion cooling, the color of the specimens was successively bluish gray, earthy yellow, dark brown, and white. Simultaneously, the number of cracks increased with the increase in the heating temperature, accompanied by material peeling.
(2) For the two cooling methods, the mass loss rate increased with the increase in temperature, and the rate of increase gradually accelerated. When the temperature was the same, the mass loss rate would gradually increase with the increase in heating temperature.
(3) The compressive strength of the PVA fiber-reinforced HSC basically decreased after the high-temperature action, and the higher the heating temperature was, the lower the compressive strength was. This is because the high temperature caused the evaporation of water in the HSC and changes to the microstructure of the concrete, which reduced the compressive strength. The maximum compressive strength would be obtained when the PVA fiber content is 0.2%.
(4) As the temperature increased, the flexural strength of the PVA fiber-reinforced HSC gradually decreased after the action of natural cooling and immersion cooling, while the flexural strength initially increased and then decreased with the increase in the fiber content. When the PVA fiber content was 0.3%, the flexural strength obviously improved. This may be because lower fiber content can fully exert the bridging effect of fibers, while excessive fibers are not conducive to the uniform distribution of the cement slurry.
(5) Through the X-ray diffraction and SEM experiments, it can be found that, at high temperatures, due to the loss of crystalline water and the decomposition of hydrates, the pores of the concrete expanded, the cracks continued to develop, and the joint between the aggregates and the cement slurry gradually separated. The diffraction intensity of CaCO_3_ showed a decreasing trend with the increase in temperature. At 800 °C, CaCO_3_ underwent decomposition and showed a gradual decrease in the mechanical strength of concrete at a macroscopic level.

It should be noted that, in the research process of this paper, high-temperature tests were conducted first, followed by mechanical performance tests. In the subsequent research, the fire resistance of the HSC under axial loads will be considered. Meanwhile, although this paper used XRD technology to analyze the composition of the HSC after the high-temperature action, the microstructure should be further analyzed to better verify the influence of the composition changes on the physical and mechanical properties.

## Figures and Tables

**Table 1:** Properties of PVA fiber.

Length (mm)	Diameter (μm)	Density (g·cm^−3^)	Tensile Strength (MPa)	Modulus of Elasticity (GPa)	Fusing Point (°C)
12	15.09	1.29	1830	40	225~230

**Table 2:** Mix proportion of PVA fiber-reinforced concrete.

PVA Fiber Content	Water (kg/m^3^)	Cement (kg/m^3^)	Fly Ash (kg/m^3^)	Slag (kg/m^3^)	Silica Fume (kg/m^3^)	Sand (kg/m^3^)	Aggregate (kg/m^3^)	Water-Reducing Agent (kg/m^3^)
0.1%	123	461.25	61.5	61.5	30.75	650.56	1070.44	4.92
0.2%	123	461.25	61.5	61.5	30.75	650.56	1070.44	4.92
0.3%	123	461.25	61.5	61.5	30.75	650.56	1070.44	4.92
0.4%	123	461.25	61.5	61.5	30.75	650.56	1070.44	4.92

**Table 3:** The number of specimens.

Temperature (°C)	Fiber Content (kg/m^3^)	Cooling Method	Test Type	Number of Specimens per Group	Total Specimens
20 °C	0, 0.1%, 0.2%, 0.3%, 0.4%	Natural cooling and immersion cooling	Compressive and flexural strength	3	60
200 °C				3	60
400 °C				3	60
600 °C				3	60
800 °C				3	60

**Table 4:** Mass loss rate of specimens during natural cooling after high-temperature action.

PVA Fiber Content%	Mass Loss Rate: AV (%), SD (%), CV (%)														
	20 °C			200 °C			400 °C			600 °C			800 °C		
	AV	SD	CV	AV	SD	CV	AV	SD	CV	AV	SD	CV	AV	SD	CV
0	0	-	-	0	-	-	0.132	0.02	0.16	2.463	0.42	0.17	4.853	0.63	0.13
0.1	0	-	-	0.133	0.04	0.28	0.200	0.03	0.14	2.692	0.32	0.12	4.899	0.69	0.14
0.2	0	-	-	0.069	0.01	0.15	0.335	0.05	0.15	2.808	0.45	0.16	4.637	0.51	0.11
0.3	0	-	-	0.066	0.01	0.15	0.332	0.04	0.11	2.935	0.41	0.14	5.014	0.75	0.15
0.4	0	-	-	0.067	0.01	0.16	0.336	0.04	0.11	3.589	0.39	0.11	5.254	0.89	0.17

**Table 5:** Mass loss rate of specimens during immersion cooling after high-temperature action.

PVA Fiber Content%	Mass Loss Rate: AV (%), SD (%), CV (%)														
	20 °C			200 °C			400 °C			600 °C			800 °C		
	AV	SD	CV	AV	SD	CV	AV	SD	CV	AV	SD	CV	AV	SD	CV
0	0	-	-	0	-	-	0.131	−0.03	0.23	-	-	-	-	-	-
0.1	0	-	-	0	-	-	0.132	−0.03	0.20	0.199	0.04	0.19	0.730	0.11	0.15
0.2	0	-	-	0	-	-	0.135	−0.03	0.22	0.497	0.07	0.15	0.849	0.12	0.14
0.3	0	-	-	0	-	-	0.002	−0.00	0.25	0.601	0.10	0.17	1.456	0.16	0.11
0.4	0	-	-	0	-	-	0.201	−0.03	0.17	0.866	0.16	0.18	1.723	0.22	0.13

**Table 6:** Measured compressive strength of specimens.

Cooling Method	Fiber Content (%)	Compressive Strength: AV (MPa), SD (MPa), CV (%)														
		20 °C			200 °C			400 °C			600 °C			800 °C		
		AV	SD	CV	AV	SD	CV	AV	SD	CV	AV	SD	CV	AV	SD	CV
Natural cooling	0	95.85	8.69	0.09	97.56	13.10	0.13	91.17	16.56	0.18	84.02	10.92	0.13	74.61	13.43	0.18
	0.1	98.64	10.50	0.11	91.17	21.61	0.24	94.50	14.72	0.16	89.46	13.42	0.15	69.57	17.39	0.25
	0.2	99.99	15.85	0.16	94.14	19.02	0.20	97.74	13.72	0.14	92.79	10.21	0.11	72.99	10.22	0.14
	0.3	99.36	11.87	0.12	92.16	20.50	0.22	93.11	16.64	0.18	84.60	14.38	0.17	66.78	9.35	0.14
	0.4	95.13	17.43	0.18	87.80	22.57	0.25	92.34	13.72	0.15	78.30	10.96	0.14	66.42	14.61	0.22
Immersion cooling	0	95.85	17.25	0.18	96.21	12.51	0.13	82.35	18.12	0.22	-	-	-	-	-	-
	0.1	98.64	14.80	0.15	90.95	24.56	0.27	95.09	19.97	0.21	81.32	16.26	0.20	77.13	9.26	0.12
	0.2	99.99	11.00	0.11	91.17	16.41	0.18	95.04	13.31	0.14	86.58	12.12	0.14	79.29	16.65	0.21
	0.3	99.36	21.86	0.22	89.28	14.28	0.16	90.86	15.45	0.17	80.28	8.83	0.11	75.33	10.55	0.14
	0.4	95.13	16.17	0.17	87.84	13.18	0.15	90.75	20.87	0.23	77.80	9.34	0.12	71.37	12.13	0.17

**Table 7:** Fitting parameters of the relationship between compressive strength and heating temperature.

Cooling Mode	PVA Fiber Content (%)	Fitting Parameter			Correlation Coefficient (Adjusted R^2^)
		*A*	*B*	*C*	
Natural cooling	0	100.7247	0.00501	−4.27698 × 10^−5^	0.97402
	0.1	97.2198	0.01824	−6.18422 × 10^−5^	0.82934
	0.2	97.3256	0.02481	−6.66056 × 10^−5^	0.84297
	0.3	97.9483	0.01021	−5.88231 × 10^−5^	0.91934
	0.4	98.2009	0.00905	−5.5638 × 10^−5^	0.86399
Immersion cooling	0	99.1690	0.04548	−1.9663 × 10^−4^	1.00000
	0.1	98.5239	−0.01722	−8.9270 × 10^−6^	0.86706
	0.2	98.9600	−0.02101	−3.79752 × 10^−6^	0.82236
	0.3	98.7259	−0.01711	−1.27116 × 10^−5^	0.85499
	0.4	98.6802	−0.01428	−1.0115 × 10^−5^	0.86318

**Table 8:** Measured flexural strength of the specimens.

Cooling Method	Fiber Content (%)	Flexural Strength: AV (MPa), SD (MPa), CV (%)														
		20 °C			200 °C			400 °C			600 °C			800 °C		
		AV	SD	CV	AV	SD	CV	AV	SD	CV	AV	SD	CV	AV	SD	CV
Natural cooling	0	6.30	0.57	0.09	6.37	0.76	0.12	5.70	0.74	0.13	4.22	0.59	0.14	-	-	-
	0.1	6.47	0.71	0.11	6.10	1.22	0.20	5.70	1.08	0.19	4.76	0.71	0.15	3.30	0.50	0.15
	0.2	0.67	1.00	0.15	6.30	1.01	0.16	6.00	1.02	0.17	4.92	0.54	0.11	3.57	0.50	0.14
	0.3	6.73	0.94	0.14	6.47	1.04	0.16	6.10	1.22	0.20	5.41	1.08	0.20	3.97	0.60	0.15
	0.4	6.57	1.12	0.17	5.90	0.77	0.13	5.67	0.85	0.15	4.67	1.07	0.23	3.10	0.37	0.12
Immersion cooling	0	6.30	0.57	0.09	6.15	1.72	0.28	5.47	0.82	0.15	-	-	-	-	-	-
	0.1	6.47	0.71	0.11	5.40	0.92	0.17	4.70	0.94	0.20	2.90	0.58	0.20	2.30	0.35	0.15
	0.2	0.67	1.00	0.15	6.00	0.96	0.16	5.00	1.05	0.21	3.60	0.76	0.21	2.70	0.38	0.14
	0.3	6.73	0.94	0.14	6.30	0.76	0.12	5.85	1.05	0.18	4.47	0.63	0.14	3.90	0.43	0.11
	0.4	6.57	1.12	0.17	5.70	1.03	0.18	4.90	0.69	0.14	3.77	0.57	0.15	2.80	0.45	0.16

**Table 9:** Fitting parameters of the relationship between flexural strength and temperature.

Cooling Mode	PVA Fiber Content (%)	Fitting Parameter			Correlation Coefficient (Adjusted R^2^)
		*A*	*B*	*C*	
Natural cooling	0	99.209	0.04150	−1.58585 × 10^−4^	0.99999
	0.1	99.076	0.00114	−7.56218 × 10^−5^	0.99157
	0.2	89.112	0.00277	−7.44423 × 10^−5^	0.98812
	0.3	98.881	0.00904	−7.2109 × 10^−5^	0.98306
	0.4	98.126	−0.00561	−7.07223 × 10^−5^	0.96345
Immersion cooling	0	99.8386	0.01021	−1.0701 × 10^−4^	1.00000
	0.1	102.028	−0.08717	1.95829 × 10^−6^	0.95602
	0.2	102.265	−0.06452	−1.7906 × 10^−5^	0.98675
	0.3	101.311	−0.03296	−2.9301 × 10^−5^	0.94413
	0.4	101.093	−0.06531	−1.0115 × 10^−5^	0.99704

## Data Availability

The original contributions presented in the study are included in the article, further inquiries can be directed to the corresponding author.
